# Numerical Study on Entropy Generation in Thermal Convection with Differentially Discrete Heat Boundary Conditions

**DOI:** 10.3390/e20050351

**Published:** 2018-05-08

**Authors:** Zhengdao Wang, Yikun Wei, Yuehong Qian

**Affiliations:** 1 Shanghai Institute of Applied Mathematics and Mechanics, Shanghai University, Shanghai 200072, China; 2 State-Province Joint Engineering Lab of Fluid Transmission System Technology, Faculty of Mechanical Engineering and Automation, Zhejiang Sci-Tech University, Hangzhou 310018, China; 3 School of Mathematical Science, Soochow University, Suzhou 215006, China

**Keywords:** entropy, Rayleigh, discrete boundary conditions, heat transfer, lattice Boltzmann method

## Abstract

Entropy generation in thermal convection with differentially discrete heat boundary conditions at various Rayleigh numbers (*Ra*) are numerically investigated using the lattice Boltzmann method. We mainly focused on the effects of *Ra* and discrete heat boundary conditions on entropy generation in thermal convection according to the minimal entropy generation principle. The results showed that the presence of the discrete heat source at the bottom boundary promotes the transition to a substantial convection, and the viscous entropy generation rate (*S_u_*) generally increases in magnitude at the central region of the channel with increasing *Ra*. Total entropy generation rate (*S*) and thermal entropy generation rate (*S_θ_*) are larger in magnitude in the region with the largest temperature gradient in the channel. Our results also indicated that the thermal entropy generation, viscous entropy generation, and total entropy generation increase exponentially with the increase of Rayleigh number. It is noted that lower percentage of single heat source area in the bottom boundary increases the intensities of viscous entropy generation, thermal entropy generation and total entropy generation. Comparing with the classical homogeneous thermal convection, the thermal entropy generation, viscous entropy generation, and total entropy generation are improved by the presence of discrete heat sources at the bottom boundary.

## 1. Introduction

The thermal convection is the fundamental physical process in a variety of engineering circumstances, such as heat accumulator system, comfortable environment, grain drying unit, electron cooling, etc. [1,2,3,4,5]. Rayleigh–Bénard (RB) convection is the simplest system of convective motion in engineering applications. Some researches about thermal convection in various fields are studied experimentally [3,4,5,6,7,8,9,10,11] or numerically [12,13,14,15,16,17,18,19,20]. It is known that when the top and bottom walls of a channel are prescribed with lower and higher temperatures, the thermal system becomes linearly unstable and convection occurs above a critical Rayleigh number, which is mainly dependent on the boundary conditions of the system and the fluid properties. However, the thermal boundary condition can be non-uniform and inhomogeneous in many applications, and the mixed thermal boundary conditions (insulating and conducting) may exist on the bottom boundary which affects the stability of natural convection [21]. Compared with the cases with homogeneous thermal boundaries, there are relatively fewer studies on natural convection with discrete thermal sources. Considering the mixed adiabatic and conductive thermal boundary condition, the system with differential heating or cooling boundaries is always unstable. Irreversible energy loss occurs in all realistic processes as a result of the mixed thermal boundary conditions. Entropy is a significant quantity for assessing the energy loss in engineering applications, and entropy generation in fluid flow and heat transfer processes can be minimized based on the second law of thermodynamics [13]. The optimized configuration of the minimum loss of available energy can be achieved in this way.

This study is significant for a series of thermal convection applications with homogeneous thermal boundary conditions. Zahmatkesh et al. [14] also reported a possible conclusion that the generation rate is high for discontinuous heating/cooling boundary. The optimal case with respect to heat transfer and entropy generation can be obtained by discontinuous heating. Oztop et al. [22] studied the thermal convection and entropy generation of a nanofluid filled cavity in magnetic field with obstacles of different shapes. Sciacovelli et al. [23] reported a critical review of entropy generation analysis on the theory and application in different types of engineering systems. Wei et al. [24]. reported the influence of different Prandtl numbers on the entropy generation in thermal convection. Jin [25] demonstrated that entropy is a powerful approach to analyze the computed results by second-law analysis. The local entropy generation rate that contains a large amount of phenomenological information are studied by many researchers, e.g., [26,27,28,29,30,31,32,33], Pizzolato et al. [26], Rejane et al. [27], Mahian et al. [28], Sheremet et al. [29], Bhatt et al. [30,31], Abbas et al. [32] and Qing et al. [33], etc.

Based on the above discussions, it is found that the various discrete thermal boundary conditions are significant on the physical mechanism and temporal–spatial characteristics of entropy generation in thermal convection. In this paper, we numerically studied the entropy generation in thermal convection process with discrete thermal boundary conditions based on the minimal entropy generation principle. The characteristics of local distributions of entropy generation are analyzed by considering the effects of *Ra* and the various discrete heat boundary conditions. The results showed that the viscous entropy generation, thermal entropy generation, and total entropy generation increase exponentially with the Rayleigh number. Comparing with the classical homogeneous thermal convection, viscous entropy generation, thermal entropy generation, and total entropy generation are intensified by the discrete heat sources at the bottom boundary.

All the numerical simulations are carried out by thermal lattice Boltzmann method (LBM). It is well known that a number of single, multi-phase, and thermal hydrodynamic problems are solved by LBM [34,35,36,37,38,39,40]. The discrete boundary conditions are easily handled due to the fully local stream-and-collide nature of LBM. The remainder of this paper is divided into the following parts. In Section 2, the thermal fluid dynamics equations and numerical method will be briefly depicted. In Section 3, the detailed results of numerical simulation and some discussions are presented. Finally, some conclusions are addressed.

## 2. Thermal Fluid Dynamics Equation and Numerical Method

### 2.1. Thermal Fluid Dynamics Equation

The classical Oberbeck–Boussinesq equations are presented as follows to study the thermal and flow physics [6,8].

(1) $$\frac{\partial \rho }{\partial t}+\nabla \cdot (\rho u)=0$$

(2) $$\frac{\partial (\rho u)}{\partial t}+u\cdot \nabla (\rho u)=-\nabla p+\nabla \cdot (2\rho \nu S)-g\beta \Delta \theta$$

(3) $$\frac{\partial \theta }{\partial t}+u\cdot \nabla \theta =\kappa \nabla^{2}\theta$$

where ν denotes the kinematic viscosity, κ denotes the diffusivity, ρ denotes the density of fluid, u denotes the macroscopic velocity, and p denotes the fluid pressure.

### 2.2. Viscosity and Thermal Entropy Generation

The viscous and thermal components of the local entropy generation rate are derived in the two-dimensional Cartesian space [30].

(4) $$S_{u}=\frac{\mu }{\theta }\left\{2\left[{\left(\frac{\partial u}{\partial x}\right)}^{2}+{\left(\frac{\partial v}{\partial y}\right)}^{2}\right]+{\left(\frac{\partial u}{\partial y}+\frac{\partial v}{\partial x}\right)}^{2}\right\}$$

(5) $$S_{\theta }=\frac{\kappa }{\theta^{2}}\left[{\left(\frac{\partial \theta }{\partial x}\right)}^{2}+{\left(\frac{\partial \theta }{\partial y}\right)}^{2}\right]$$

The total entropy generation rate is represented by the summation of the above two terms

(6) $$S=S_{u}+S_{\theta }$$

### 2.3. Numerical Method for Thermal Fluid Dynamics Equation

The lattice Boltzmann equation for the fluid flow field is [36,37,38].

(7) $$f_{i}(x+c_{i}\Delta t,t+\Delta t)=f_{i}(x,t)+(f_{i}^{eq}(x,t)-f_{i}(x,t))/\tau_{\nu }+F_{i}$$

The lattice Boltzmann equation for the temperature field is given by the following equation

(8) $$g_{i}(x+c_{i}\Delta t,t+\Delta t)=g_{i}(x,t)+(g_{i}^{eq}(x,t)-g_{i}(x,t))/\tau_{\theta }$$

where $f_{i}(x,t)$ and $g_{i}(x,t)$ are the density and temperature distribution functions at (x,t), $c_{i}$ is the discrete velocity. $F_{i}$ denotes the discrete force term in Equation (7), $\tau_{\nu }$ and $\tau_{\theta }$ denote the relaxation times for density evolution equation and temperature evolution equation in lattice Boltzmann method. The equilibrium functions for the density and temperature distribution functions are presented by the following equations [37], respectively.

(9) $$f_{i}^{eq}=\rho w_{i}[1+\frac{c_{i}\cdot u}{c_{s}^{2}}+\frac{{\left(c_{i}\cdot u\right)}^{2}}{c_{s}^{2}}-\frac{u^{2}}{2c_{s}^{2}}]$$

(10) $$g_{i}^{eq}=\theta w_{i}[1+\frac{c_{i}\cdot u}{c_{s}^{2}}+\frac{{\left(c_{i}\cdot u\right)}^{2}}{c_{s}^{2}}-\frac{u^{2}}{2c_{s}^{2}}]$$

where $w_{i}$ denotes the weight coefficient [38]. The kinematic viscosity ν and the diffusivity κ are computed by the mesoscopic method through the following equations

(11) $$\nu =\frac{2\tau_{\nu }-1}{6}\frac{{\left(\Delta x\right)}^{2}}{\Delta t},\;\kappa =\frac{2\tau_{\theta }-1}{6}\frac{{\left(\Delta x\right)}^{2}}{\Delta t}.$$

The density, macroscopic velocity, and temperature are given by following equations

(12) $$\rho =\sum_{i=0}^{8}f_{i},\;\rho u=\sum_{i=0}^{8}c_{i}f_{i},\;\theta =\sum_{i=0}^{8}g_{i}.$$

The formulations of density, momentum and temperature are derived using the Chapman–Enskog expansion. A macroscopic length scale (*x*_1_ = *εx*) and two macroscopic time scales (*t*_1_ = *εt*, *t*_2_ = *εt*) are employed to obtain the classical Oberbeck–Boussinesq equations (Equations (1)–(3)). As two time scales ∂*t* = *ε*∂*_t_*_1_ + *ε*^2^∂*_t_*_2_ and one spatial scale ∂*_x_* = *ε*∂*_α_* are carried out for the Frisch, Hasslacher and Pomeau (FHP) model. The inertial terms in the classical Oberbeck–Boussinesq equations (Equation (7)) can be reproduced by executing the streaming step using the above Chapman–Enskog expansion.

**Figure 1:**
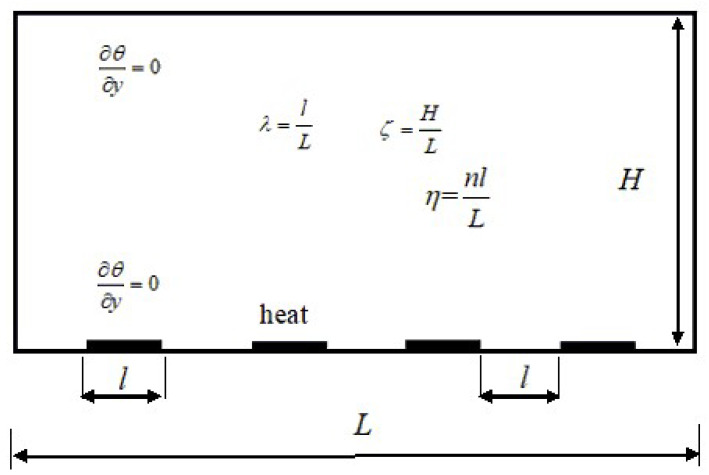
Computational geometry and boundary conditions.

The typical geometrical schematic is described in Figure 1. The inhomogeneities are restricted only to the bottom plate (*y* = 0), and are made of alternating regions of either isothermal boundary condition, *θ* = *θ*_down_, which is denoted by black regions, or adiabatic boundary condition, ∂*_y_θ* = 0. The upper boundary (*y* = *H*) is kept at constant temperature, *θ* = *θ*_up_. In this simplified geometry, the width to height ratio of the cavity, *ξ* = *H*/*L*, and other two new dimensionless parameters are used to define the geometrical configuration of the heating boundary, namely the percentage of single heat source area *λ* = l/*L*, and the total percentage of heating source area, *η* = *nl*/*L*, in Figure 1, where *n* is the number of heat source and *l* is the length of single heat source region. In the limiting case of *η* = 1, the model recovers to the usual homogeneous RB convection. The quantities *λ* and *η* are important factors for discrete heat source boundary conditions. It is easy to understand the changes with different *λ* at fixed *η* from an applied point of view.

The Rayleigh number is the most significant dimensionless parameter in the thermal convection which is given by the following expression.

(13) $$Ra=\beta \Delta \theta gH^{3}/\nu \kappa$$

The *Nusselt* number is a dimensionless parameter in the thermal convection to reflect the heat transfer performance of the system. It is computed in LBM by the following expression.

(14) $$Nu=1+\langle u_{y}\theta \rangle /\kappa \Delta \theta H$$

where Δθ denotes the difference of temperature between the top wall and the bottom wall, H represents the height of channel, $u_{y}$ represents the velocity in vertical direction, and 〈⋅〉 denotes the average value of the entire domain.

In general, the accuracy and stability of the LBM is closely related with the boundary conditions, which play key roles in LBM. The non-equilibrium extrapolation approach and the periodic condition are implemented in this paper. The idea of non-equilibrium extrapolation approach is given by the following expressions

(15) $$f_{i}(x_{b},t)=f_{i}^{eq}(\rho_{w},u_{w})+(f_{i}(x_{f},t)-f_{i}^{eq}(\rho_{f},u_{f}))$$

(16) $$g_{i}(x_{b},t)=g_{i}^{eq}(\rho_{w},u_{w})+(g_{i}(x_{f},t)-g_{i}^{eq}(\rho_{f},u_{f}))$$

In which the non-equilibrium contribution is derived from the fluid node $x_{f}$ next to $x_{b}$ along the boundary normal vector. During propagation, the unknown incoming populations on one side are derived by those leaving the domain at the opposite side. The idea of the periodic condition approach is presented by the following expressions

(17) $$f_{i}(x,t)=f_{i}(x+L,t)$$

(18) $$g_{i}(x,t)=g_{i}(x+L,t)$$

where the vector **L** denotes the periodicity direction and length of the flow pattern.

## 3. Results and Discussion

In the following section, the parameters *λ* and *η* are selected at different values to study their effects in low Rayleigh number regime. For thermal convection with discrete heat boundary conditions at different *Ra*, *λ*, and *η*, it is supposed that the Boussinesq approximation is applied for the incompressible fluid, and two-dimensional characteristics are presented in Figure 1. The no-slip boundary conditions are employed for top and bottom walls. Periodic boundary conditions are implemented at vertical boundaries in all numerical simulations. The dimensionless temperature of discrete heat source equals to 0.5 in Figure 1, and the dimensionless initial temperature of the fluid is −0.5. All numerical simulations at different *Ra*, *λ* and *η* are performed using LBM in two-dimensional thermal convection, and 540 × 270 lattices are used. The *Prandtl* number is defined as *Pr* = *ѵ*/*κ* and is fixed at 0.71.

**Figure 2:**
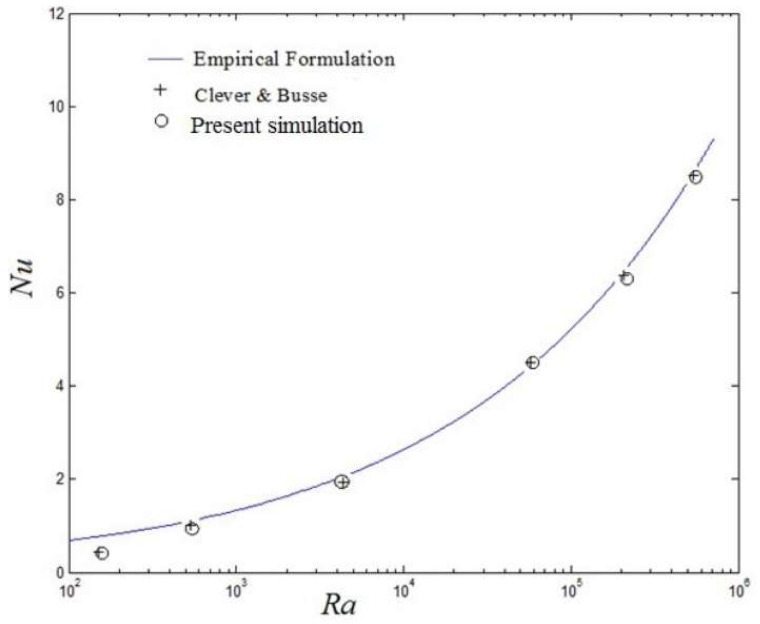
Nusselt number *Nu* as a function of Rayleigh number in traditional Rayleigh–Bénard (RB) convection for homogeneous boundary conditions (*η* = 1).

The Nusselt number is computed and compared with the result of Clever and Busse [41] to validate the code. Figure 2 presents the relationship between the Nusselt number *Nu* and the Rayleigh number *Ra* for classical homogeneous thermal convection (*η* = 1). The blue curve is the empirical expression *Nu* = 1.56 (*Ra*/*Ra_c_*)^0.296^, and the circle symbols denote the results of the present simulations using LBM, and the plus symbols represent the numerical results obtained by Clever and Busse [41]. It is seen that the value of the *Nusselt* number obtained using the present LBM is quite consistent with the theoretical value and benchmark solutions, which validates the accuracy of the numerical simulations by the LBM.

**Figure 3:**
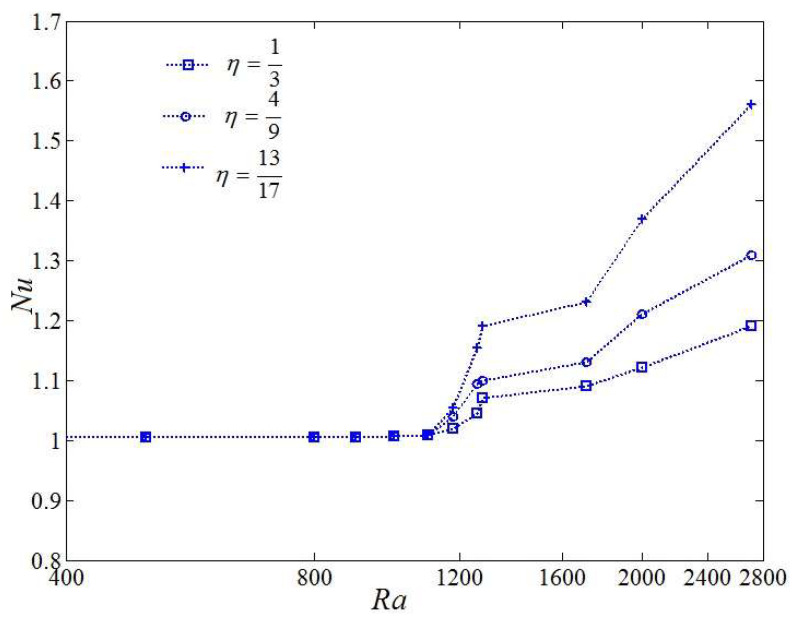
Nusselt number with changing Rayleigh number and with discrete heat boundary conditions for different *η*.

Figure 3 shows the variation of Nusselt number with Rayleigh number for discrete heat boundary conditions at various *η*. The definition in Equation (14) achieves the condition that the magnitude of Nusselt number approaches to *Nu* ≈ 1 for any *η* as the Rayleigh number is less than 1200. The final steady-state (*t* → ∞) magnitude of the Nusselt number at different Rayleigh numbers is represented by the three different cases in Figure 3. As the Rayleigh number is less than 1707, the Nusselt number gets close to *Nu* ≈ 1 for the classical homogeneous RB convection [37]. However, it is noticed that comparing with the classical homogeneous RB convection, the introduction of the discrete heat sources at the bottom boundary promotes the transition to thermal convection.

### 3.1. Analysis of Flow and Temperature Field

**Figure 4:**
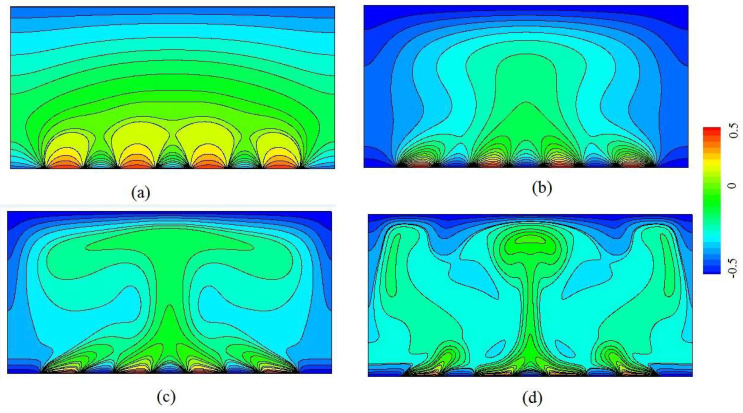
Temperature distributions (isotherms) at various Rayleigh number from (**a**) to (**d**). (**a**) *Ra* = 10^3^; (**b**) *Ra* = 10^4^; (**c**) *Ra* = 10^5^; (**d**) *Ra* = 10^6^.

Steady-state isotherms of RB convection with discrete heat boundary conditions are present in Figure 4 for *η* = 4/9, *λ* = 1/9, *ξ* = 1/2, at *Ra* = 10^3^, 10^4^, 10^5^ and 10^6^. As shown in Figure 4, the local hot fluid close to each heat source moves upward in an independent way and the isotherms merge at the top region of the channel at *Ra* = 10^3^. However, the cold fluid close to the cold sources moves upward and increases the temperature near every cold source. It is seen that with increasing Rayleigh number, the heat sources effectively heat the fluid from the central portion of the channel to the top wall. The hot fluid flow moves upward and the cold fluid moves downward near the top wall flow, while the temperature decreases near the side boundaries. Two trends with increasing Rayleigh number are found for the isotherm distribution. The mixing of the cold and hot fluids is enhanced, and an increased temperature gradient is found in the region close to the bottom and top boundaries. It is concluded that the presence of the discrete heat sources leads to the enhanced heat transfer in the channel.

**Figure 5:**
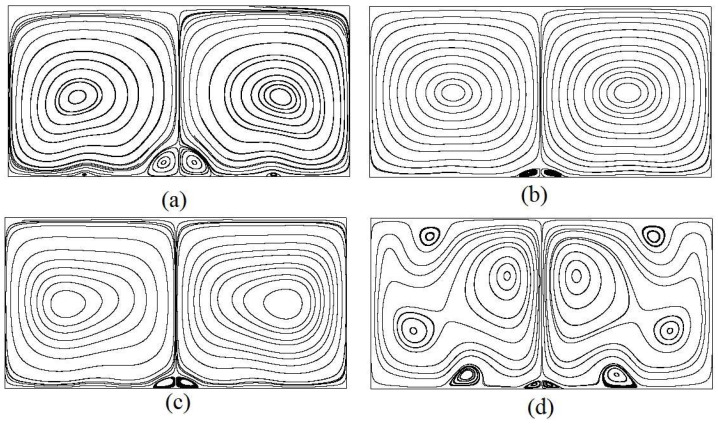
Streamlines of the thermal convection flow at various Rayleigh number from (**a**) to (**d**). (**a**) *Ra* = 10^3^; (**b**) *Ra* = 10^4^; (**c**) *Ra* = 10^5^; (**d**) *Ra* = 10^6^.

Typical steady-state flow patterns of RB convection are shown in Figure 5. It is observed that for *Ra* = 10^3^, two large symmetrical vortices appear in the central region of channel as the typical behaviors of the heated flow; two symmetrical secondary vortices appear in the central region of the bottom wall, and small vortices appear between the heat sources on the bottom wall. The vortices gradually expand to be of elliptic shape with increasing *Ra*, and move towards both vertical sides of the channel. As *Ra* reaches to 10^6^, the two vortices break up into multiple vortices.

### 3.2. Analysis of S_u_ and S_θ_

**Figure 6:**
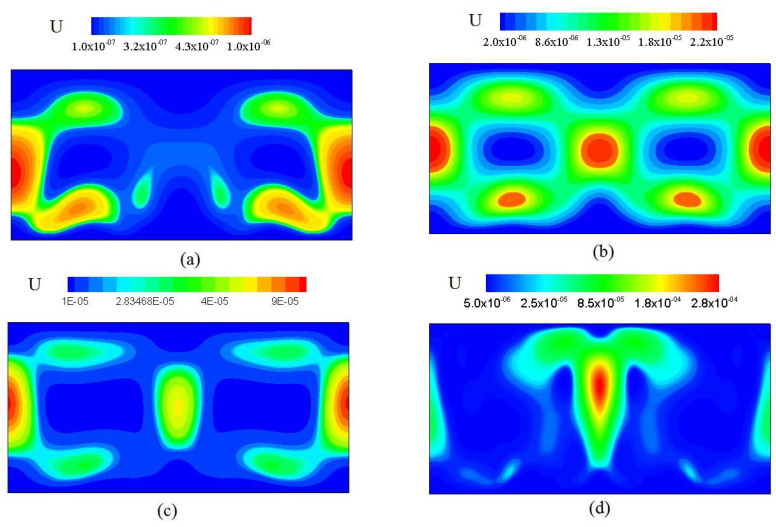
Velocity distribution of the thermal convection flow at various Rayleigh number from (**a**) to (**d**). (**a**) *Ra* = 10^3^; (**b**) *Ra* = 10^4^; (**c**) *Ra* = 10^5^; (**d**) *Ra* = 10^6^.

**Figure 7:**
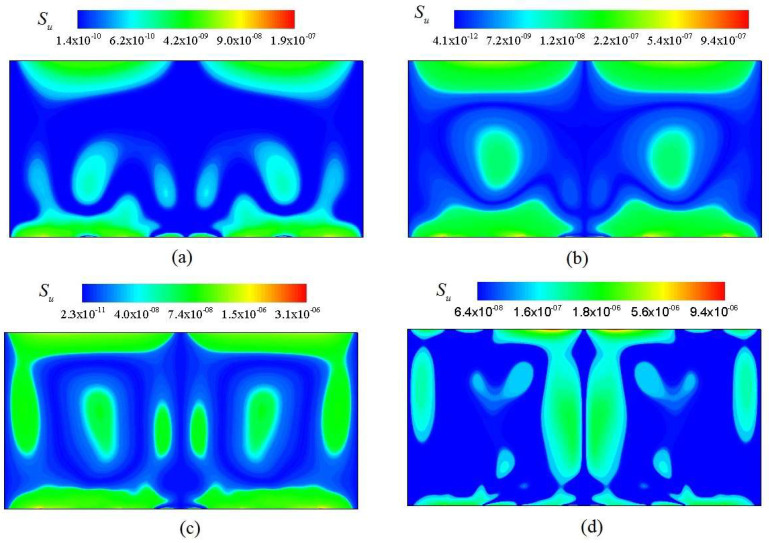
Viscous entropy generation rate at various Rayleigh number. (**a**) *Ra* = 10^3^; (**b**) *Ra* = 10^4^; (**c**) *Ra* = 10^5^; (**d**) *Ra* = 10^6^.

Figure 6 shows the distribution of velocity isolines at *Ra* = 10^3^, 10^4^, 10^5^ and 10^6^. The viscous entropy generation rate at four *Ra* ispresented in Figure 6a–d. As shown in Figure 6 and Figure 7, the significant *S_u_* mainly clusters in the region with steepest velocity gradient. It is concluded that with increasing *Ra*, the significant *S_u_* gradually propagates to the central region of the channel, which mainly occurs in the region with largest velocity gradient in the majority portion of the channel.

**Figure 8:**
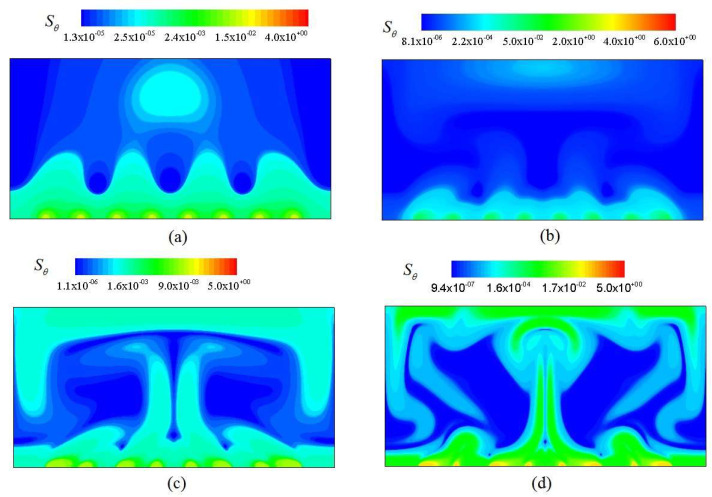
Thermal entropy generation rate at various Rayleigh number from (**a**) to (**d**). (**a**) *Ra* = 10^3^; (**b**) *Ra* = 10^4^; (**c**) *Ra* = 10^5^; (**d**) *Ra* = 10^6^.

Figure 8 shows the thermal entropy generation rate *S_θ_* at four *Ra*. From Figure 4 and Figure 8, it is seen that the significant *S_θ_* clusters on the interfaces between hot and cold fluids, which is mainly originated from the largest temperature gradient near wall regions. As shown in Figure 8b–d, the significant *S_θ_* constantly propagates to the central region of the channel, which is closely related with the largest temperature gradient in the majority of the channel.

**Figure 9:**
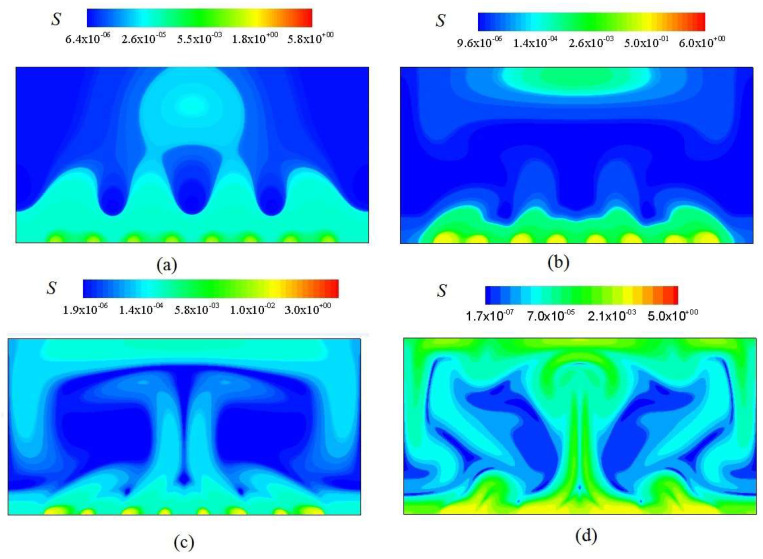
Total entropy generation rate at various Rayleigh number from (**a**) to (**d**). (**a**) *Ra* = 10^3^; (**b**) *Ra* = 10^4^; (**c**) *Ra* = 10^5^; (**d**) *Ra* = 10^6^.

The corresponding total entropy generation rate *S* at four *Ra* is presented in Figure 9. The distribution of *S* is consistent with that of *S_θ_* at the same *Ra*. It is shown that the heat transfer plays a leading role on the flow in the channel. Comparing Figure 7 and Figure 8, it is seen that *S_θ_* is much larger in magnitude than *S_u_*. It is also indicated that the heat transfer irreversibility plays a leading role in the entropy generation of thermal convection.

**Figure 10:**
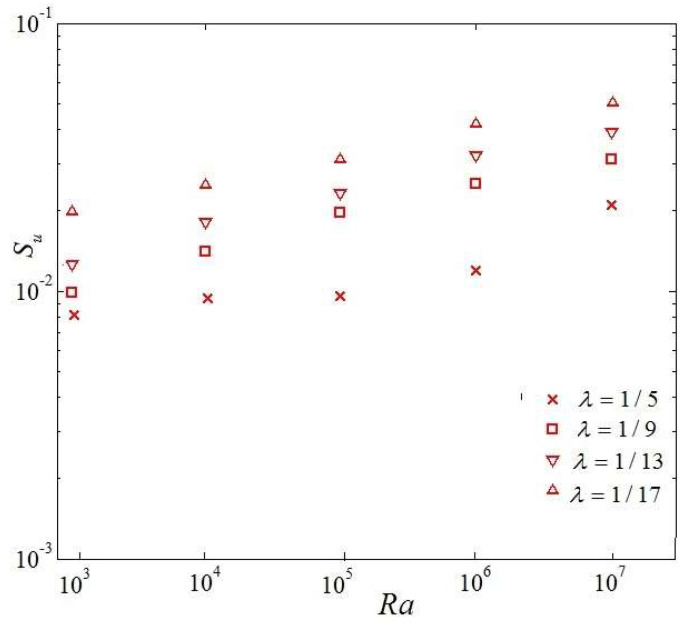
Variety of viscous entropy generation owing to effects vs. Rayleigh number.

The effect of single heat source area at the bottom boundary (*λ*) on thermal entropy generation, the viscous entropy generation, and total entropy generation is investigated in the following section. The percentage of single heat source area at the bottom boundary indicates that, with a smaller percentage of single heat source area, the number of the discrete heat boundary sources increases. Figure 10 shows the variation of entropy generation owing to viscous effects for four aspects ratios (*λ*) and four irreversibility coefficients versus Rayleigh number. It is observed that with the increase of the Rayleigh number, the viscous entropy generation increases exponentially. One can also see that with smaller percentages of single heat source area at the bottom boundary, the viscous entropy generation increases in magnitude. It is obtained that the variation of entropy generation is related with the viscous effects that resulted from the velocity gradients in this case.

**Figure 11:**
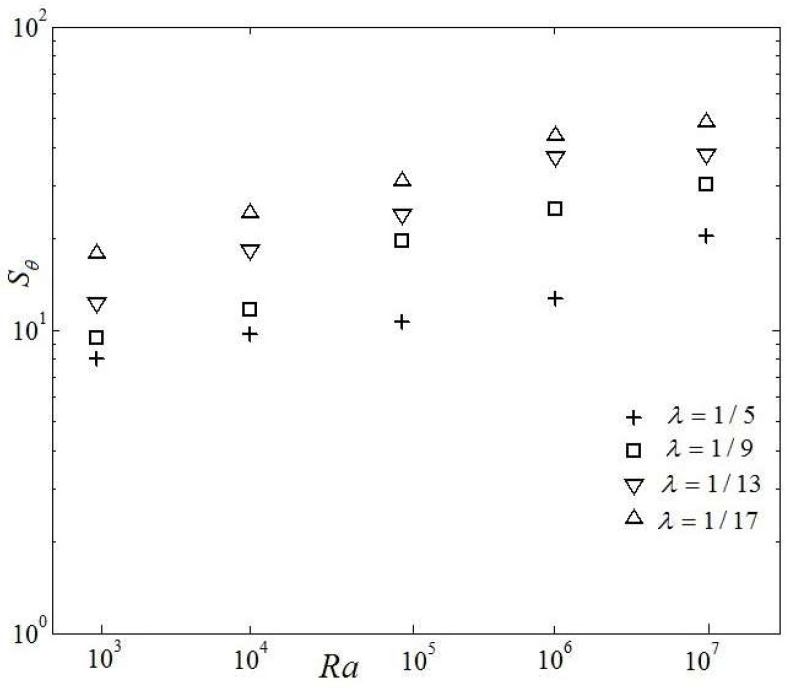
Variety of thermal entropy generation owing to effects vs. Rayleigh number.

Figure 11 shows the variation of entropy generation considering thermal effects for four *λ* and four irreversibility coefficients versus Rayleigh number. It is seen that the thermal entropy generation increases exponentially with the increase of Rayleigh number. One can also see that the smaller percentage of single heat source area at the bottom boundary leads to the larger magnitude of thermal entropy generation. This feature is closely related with the variation of thermal boundary layer near every heat source and to the characteristics of entropy generation process. It is noted that the influence of entropy generation is closely related with the thermal effects which are resulted from the thermal gradients as derived by its definition.

**Figure 12:**
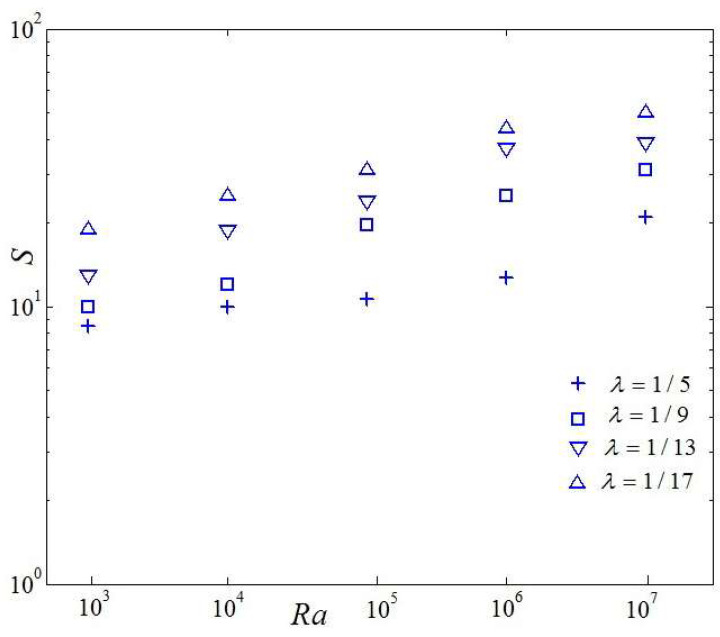
Variety of total entropy generation owing to effects vs. Rayleigh number.

Figure 12 demonstrates the variation of total entropy generation for five *λ* and four irreversibility coefficients vs. Rayleigh number. It is seen that the magnitude and variation pattern of total entropy generation are consistent with the thermal entropy generation. Based on detailed comparisons between Figure 10 and Figure 11, one can also see that the magnitude of the thermal entropy generation is about two orders of magnitude that of the viscous entropy generation. The thermal entropy generation plays a key role in the heat transfer irreversibility. Meanwhile, it is seen that comparing with the classical homogeneous RB convection (*λ* = 1), the thermal entropy generation, viscous entropy generation, and total entropy generation are improved owing to the presence of the discrete heat sources at the bottom boundary.

## 4. Conclusiuons

In this paper, by changing the Rayleigh number and thermal surface ratio at the bottom boundary, the entropy generations are investigated by lattice Boltzmann method. Several conclusions are derived.

Our results mainly indicate that the transition to bulk convection is improved by the discrete heat boundary. It is found that the critical Rayleigh number for transition is between 1150 and 1230 for the discrete heat boundary. The thermal entropy generation, viscous entropy generation, and total entropy generation increase exponentially with the Rayleigh number. It is demonstrated that with the increase of *Ra*, it becomes significant that the viscous entropy generation gradually propagates to the central region of the channel, which mainly occurs in the region with steepest velocity gradient in the majority of the channel. The influence of entropy generation is related to the thermal effects considering the thermal gradients. Thermal entropy generation plays a key role in the flow heat transfer irreversibility, frictional irreversibility can be neglected in thermal convection with differentially discrete heat boundary. One can also obtain that the smaller percentage of single heat source area at the bottom boundary increases the magnitudes of viscous entropy generation, thermal entropy generation, and total entropy generation. Comparing with the classical homogeneous thermal convection, the presence of the discrete heat sources at the bottom boundary improves the thermal entropy generation, viscous entropy generation, and total entropy generation.

It is observed in this study that the hydrodynamic and thermal problems are highly coupled. The differentially discrete heat boundary conditions with larger aspect ratios are the better option for thermal convection in a thermophysical configuration. The system efficiency is enhanced with the increase of the heat aspect ratio in the discrete heat bottom boundary. This type of discrete heat boundary condition and thermophysical configuration are extensively applied in many kinds of equipment, such as the absorber plate of a solar thermal collector, or the existing plates of electronic circuit.
